# University Students’ Perceptions and Experiences of a Suicide Prevention Gatekeeper Program (GUIAS_Program): Improvements and Implications for Education

**DOI:** 10.1111/hex.70599

**Published:** 2026-02-18

**Authors:** Patricia García‐Pazo, Paula Nadal Canet, Margalida Miro‐Bonet, Elena Gervilla García

**Affiliations:** ^1^ Department of Nursing and Physiotherapy University of the Balearic Islands Palma Spain; ^2^ Care, Chronicity, and Health Evidences Research Group (CurES) Health Research Institute of the Balearic Islands (IdISBa) Palma Spain; ^3^ Department of Psychology University of the Balearic Islands Palma Spain; ^4^ Statistical and Psychometric Procedures Applied in Health Science (Psicomest) Health Research Institute of the Balearic Islands (IdISBa) Palma Spain

**Keywords:** clinical simulation, gatekeepers, qualitative research, suicide, suicide prevention, university students

## Abstract

**Introduction:**

Suicide prevention in university settings presents a significant public health challenge due to the high prevalence of suicidal ideation and behaviours among students. This qualitative study explores the perceptions and experiences of students who participated in the *Universtiy Manager in Suicide Identification and Care* (in Spanish, GUIAS: *Gestores Universitarios en Identificación y Atención al Suicidio*) training programme, designed to equip them with skills for early detection and initial management of at‐risk situations.

**Method:**

An ethnomethodological thematic analysis was conducted using self‐recorded semi‐structured interviews. Participants were undergraduates enroled in the 2023–2024 academic year at the University of the Balearic Island, studying degrees in Nursing, Medicine, Psychology, Physiotherapy, Social Work, or Pedagogy. A total of 21 students submitted interviews; however, theoretical data saturation was reached after analysing 15.

**Result:**

Thematic analysis identified four key themes: (a) satisfaction with programme design, (b) satisfaction with content, (c) achievements and expectations, and (d) suggestions for improvement. Participants valued the course's mixed‐method approach, which combined theoretical modules, clinical simulations, and reflective activities, fostering a flexible and personalised learning experience. Clinical simulations were particularly highlighted for their role in developing practical skills.

**Conclusion:**

Findings indicate that the programme effectively supports competence development in suicide prevention. Participants recommended broadening content and optimising dissemination strategies to enhance the programme's reach and impact. These insights underscore the importance of refining such initiatives within university settings to strengthen suicide prevention efforts and improve student preparedness in handling at‐risk situations.

**Patient or Public Contribution:**

Individuals with lived experience of suicidal crises contributed to the design and evaluation of the programme, providing their perspective to ensure that the content was aligned with the real needs of the university community. Additionally, a student participated as co‐author, which allowed for a more contextually‐appropriate adaptation of the training programme to its intended recipients.

## 1 Introduction

Suicide prevention is a pressing global challenge and a public health priority, particularly among the university population, where rates of suicidal ideation and behaviour are alarming. In Spain, 9.2% of university students reported suicidal ideation in the past year, and 24% have experienced it at some point in their lives [1, 2]. International studies suggest that prevalence rates range from 14% to 24%, influenced by sociocultural and institutional contexts, including factors such as social isolation, academic pressure, and financial difficulties [3].

Suicide is one of the leading causes of mortality among young university students, a particularly vulnerable group due to the multiple challenges associated with this transitional stage. A recent scoping review highlighted the close association between suicidal behaviour and the stigma surrounding help‐seeking [4]. Moreover, Avanci and Gonçalves [5] identify academic stress and loneliness as common triggers, while experiences of cultural or racial discrimination significantly increase the risk of suicidal ideation in certain groups [6].

The COVID‐19 pandemic further exacerbated these issues, heightening levels of anxiety and depression, which are predisposing factors for suicide [7]. This aligns with previous studies underscoring the need for tailored and culturally sensitive interventions for this population [1, 2].

Universities represent a strategic environment to address this issue, as they can provide accessible, effective, and evidence‐based preventive programmes, mental health resources, and social support. However, to ensure their efficacy, these initiatives must be grounded in robust scientific evidence and consider the specific needs of students, as highlighted in the *Plan de Acción Integral Sobre Salud Mental 2013–2030* [8].

Noteworthy approaches include gatekeeper training, which prepares individuals to identify suicide risk signs and connect at‐risk individuals with specialised services [9, 10]. The effectiveness of suicide prevention programmes through gatekeeper training in university contexts has been well‐documented, with positive outcomes in the identification of individuals at risk, reduction of suicidal ideation, and suicide prevention [10]. Programmes such as QPR (Question, Persuade, and Refer) [11], I CARE, or PRECEDE‐PROCEED [12] have shown promising results in reducing both suicidal ideation and attempts. Similarly, the YAM [Youth Aware of Mental Health] programme [13] has had a positive impact in adolescent populations. These interventions reinforce the idea that a combination of education, awareness‐raising and practical skill‐building can make a significant difference in suicide prevention [9].

These approaches have been successfully adapted to various educational contexts, including higher education, vocational training, and secondary and primary education [12, 13, 14], as well as to diverse cultural contexts, demonstrating a measurable impact on reducing suicide risk in various populations [15].

Regarding teaching methodology, techniques such as clinical simulation [16] and role‐playing—used in the YAM programme—stand out as effective tools for training non‐technical preventive skills. These techniques offer a safe and structured learning environment in which students can apply theoretical knowledge, develop cognitive and emotional competences, and gain confidence to intervene in real‐life situations [15, 17].

This study focuses on the GUIAS (*University Manager in Suicide Identification and Care*) training programme, designed to train university students in the identification of risk signals and early intervention as gatekeepers. The adaptation of the programme to address the specific needs, contexts and life experiences of university students is crucial to ensure its effectiveness.

The evaluation of students’ perceptions of these programmes is critical to their success and sustainability. Recent research has shown that initiatives promoting open dialogue on mental health and providing practical, skills‐based learning are highly valued by young people [18, 19]. Moreover, extracurricular programmes, such as suicide prevention volunteer activities, have had a positive impact by transforming attitudes, values, and beliefs, and fostering a more informed and empathetic perception of mental health [20].

### 1.1 Aim for This Study

The main aim of the current study is to investigate university students’ perceptions and experiences concerning the 'University Manager in Suicide Identification and Care' (GUIAS) training programme. Specifically, the research seeks to evaluate participants’ satisfaction with the training provided, assess achievements and address unmet expectations. Drawing upon these findings, the study aspires to identify areas for enhancement in order to optimise future iterations of the programme.

## 2 Methods

### 2.1 Design and Study Setting

We conducted an ethnomethodological design of thematic analysis. Ethnomethodology is a sociological approach that aims at revealing the nature of social orders through the study of how people try to make sense of the world around them through words and actions in their stories [21]. This enables a deeper understanding of the participants’ perceptions, meanings, and experiences regarding the training received.

The study was conducted at the University of the Balearic Islands (UIB), founded in 1978 and located in Palma, the capital of Mallorca. The university also has campuses in Menorca and Ibiza. In the 2023–2024 academic year, the UIB had a total enrolment of 15,789 students, including 11,926 undergraduate students, 1479 postgraduate students, and 930 doctoral candidates. The UIB offers degree programmes in health sciences (Nursing, Psychology, Physiotherapy, and Medicine), social and human sciences, and other disciplines.

At the University of the of the Balearic Islands, we developed the first comprehensive training programme for suicide prevention during the 2022–2023 academic year, which was implemented in 2024. This course combines theoretical and practical modules that must be completed sequentially. It aims to provide students with theoretical knowledge about suicide, practical skills to deal with individuals at risk of suicide, and criteria for referral to support resources based on risk levels. The programme has been accredited by UIB academic committee, granting two European Credit Transfer and Accumulation System (ECTS) credits, equivalent to 50 h of instruction.

The course uses diverse pedagogical methodologies, including textual presentations of theoretical content, educational videos, infographics, and synchronous online sessions (webinars) to clarify and consolidate knowledge. Additionally, clinical simulation with a standardised patient (an actress) is employed to strengthen non‐technical skills essential for the initial management of individuals at risk of suicide.

The curriculum is divided into six mandatory modules and one optional module focused on emotional regulation (Table 1). These modules are delivered virtually. Additionally, there are two mandatory webinars conducted by external expert instructors and one mandatory in‐person clinical simulation session. During these sessions, students put into practice their acquired knowledge of initial crisis management in suicide scenarios.

**Table 1:** Description of modules, content, and learning methodology.

Module	Title	Content	Methodology
1	Introduction	Epidemiology, Contextualisation, Myths	Text (PDF), Infographics, Short Educational Videos
2	Basic Concepts	Types of suicidal behaviour, Risk factors, Protective factors, Warning signs	Text (PDF), Educational Videos
3	Initial Management	Warning signs, Suicide risk assessment (detection tools), Initial management [dos and don'ts, safety plan]	Text (PDF), Educational Videos, Didactic Infographics
4	Support Resources	Institutional resources (IBSALUD), Community resources in the Balearic Islands	Educational Videos
5	Communication and Suicide	Werther Effect and Papageno Effect, WHO recommendations on reporting suicide‐related deaths	Text (PDF), Infographics, Educational Videos
6	Postvention Survivors	Survivors, Grief, Intervention areas, Postvention	Text (PDF), Educational Videos

### 2.2 Participants

The participants were undergraduate students enroled in the 2023–2014 academic year in various degree programmes at the University of the Balearic Islands. A total of 54 students initially enroled in the A training program, of whom 44 successfully completed the course. Ultimately, 21 students submitted self‐recorded interviews. However, theoretical data saturation was reached after analysing 15 interviews. In accordance with the methodological guidance [22, 23], a sequential analysis of three additional interviews was conducted to assess the attainment of theoretical saturation The participants were students from the degree programmes in Nursing, Medicine, Psychology, Physiotherapy, Social Work, and Pedagogy.

The research team contacted students via email to provide them with access credentials for the course. At the beginning of the programme, students watched an introductory video in which instructors presented the course objectives and contents, as well as research goals.

A purposive theoretical sampling method was employed. Recruitment was conducted through study‐related information shared on the course digital platform. Participation in the interviews was voluntary, anonymous, and confidential.

Regarding the sample characteristics, 52.38% of participants were Psychology students, 38.09% were Social Education students, and 4.7% were enroled in Nursing and Physiotherapy programmes. The majority of participants in the GUIAS programme were female (85.71%), with an average age of 21.48 years. Inclusion criteria required participants to have successfully completed the A training programme and achieved a passing grade. No financial compensation was provided for participation.

### 2.3 Data Collection

Twenty‐one self‐recordings were compiled based on a semi‐structured interview guide (Table 2). The interview script was designed by the authors of the article and pilot‐tested with a student. The interview method is widely used in health and social sciences research, as it enables a deeper understanding of participants’ experiences and perspectives, ensuring alignment with the study's research question [25].

**Table 2:** Interview guide: open‐ended questions.

Interview questions
1. What are your thoughts on this training programme? Did you find it satisfactory? 2. How do you perceive the design and structure of the course? 3. Were your expectations met? 4. Which expectations were not met? 5. What improvements would you suggest? What aspects would you expand on, modify, or remove?

The data collection process took place between April and July 2024, coinciding with the completion of the GUIAS training programme during the second semester of the 2023/2024 academic year. At the end of the course, participants were invited to record an audio file responding to a set of open‐ended interview questions (Table 2). The recordings were submitted through the university's digital learning platform or via email to the research team. A total of 21 students submitted their self‐recorded interviews, with all responses collected anonymously to ensure participant confidentiality. The self‐recorded interviews had an approximate duration of 2–5 min.

The recorded interviews were transcribed using the Microsoft Word dictation tool and subsequently anonymized and analysed in the order they were received. Transcripts were coded sequentially and assigned anonymized identifiers (STD 1, 2, 3…) to preserve participant anonymity. All interviews were conducted in Spanish and later translated into English by bilingual researchers. Translations were subsequently reviewed by a native English philologist. Accuracy was ensured through back‐translation and team consensus. This approach allowed for the systematic organisation of responses and facilitated the ensuing data analysis.

### 2.4 Data Analysis and Rigor

Thematic analysis facilitated the identification of recurring patterns and themes in the collected data, ensuring a systematic and rigorous interpretation of participants’ experiences [24, 25]. A thematic analysis was conducted following a systematic, multi‐phase approach. First, the researchers familiarised themselves with the data through repeated readings. Subsequently, initial codes were generated inductively, followed by the identification and narrowing down of themes or categories. These themes or categories were then defined to ensure theoretical coherence with the study's specific objectives.

To enhance rigor and reliability, researcher triangulation and theoretical data saturation were employed. Three researchers (PG, MM and EG) independently coded the data. Discrepancies were discussed and resolved to establish consensus in multiple meetings. This iterative process contributed to increase the analytical depth and validity of the findings. The analysis reached theoretical data saturation when no new themes or insights emerged, ensuring the completeness of the findings.

For this article, we have reviewed the items of the Consolidated Criteria for Reporting Qualitative Research (COREQ) to guarantee the quality of the study and enhance transparency in the report, obtaining a score of 86.20% [26].

### 2.5 Reflexivity Statement

The research team comprises authors with diverse disciplinary backgrounds and varying perspectives on the investigated phenomenon. This interdisciplinarity enriched the data analysis with diverse and plural viewpoints. All the researchers are women. Three of the authors (PG, MM and EG) are experts in health and behavioural sciences. PN, a third‐year Nursing undergraduate trained as a GUIAS, added student insights into the research, providing a deeper and more meaningful understanding of the data and analysis. Finally, MM is an expert in qualitative health research, PG specialises in suicidal behaviour, and EG is an active researcher in prevention science, serving as president of the European Society for Prevention Research (EUSPR).

## 3 Results

**Figure 1:**
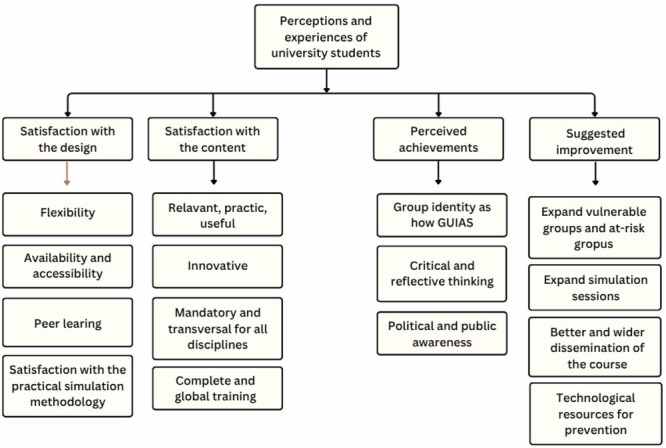
Tree diagram of emergent categories and codes.

The thematic analysis revealed four categories: (a) satisfaction with the design, (b) satisfaction with the content, (c) achievements and expectations, and (d) suggestions for improvement. These categories provide a comprehensive and integrated understanding of students’ learning experience (Figure 1). Each of these categories is described in detail below. Given the limited length of the article, they are illustrated with one or, at most, two quotes. The quotes in Spanish, the original language of the interviews, can be found in the Supporting Information S1.

### 3.1 Satisfaction With the Course Design

This category encompasses students’ opinions and experiences regarding how the course was organised and presented. Specifically, it includes references to the structure, visual design, methodology, and assessment strategies.

One of the key aspects highlighted is the flexibility of the course, which facilitates its integration into students’ schedules and compatibility with other activities. The course design caters for diverse learning paces, timings, and rhythms:

It's very positive that you can complete the different modules whenever and wherever you want

(STD8)

The course design respects individual learning times

(STD16)

Students also express satisfaction with the accessibility and availability of materials, which enables them to revisit content as often as necessary and at any time:

You can consult it as many times as you want, watch the videos multiple times

(STD1)

The discussion forum is considered highly valuable by participants, as it allows them to share opinions and questions both with peers and course instructors:

You can share doubts in the forum, with those responsible for the course, and with classmates

(STD1)

Among the methodologies employed during the course, clinical simulation is particularly appreciated. Students regard it as a practical tool that enhances their understanding of theoretical content and provides a realistic perspective on suicide:

I found it very practical to see these concepts applied in clinical simulation

(STD4)

It brings realism to all the theoretical content

(STD12)

### 3.2 Satisfaction With the Content

This category encompasses all references to the materials, including themes, concepts, case studies, and supplementary resources that facilitated learning.

Students expressed high satisfaction with the topics and activities provided, describing them as comprehensive, relevant, practical, and useful to achieve the training objectives. They emphasised that none of the content was irrelevant or dispensable:

The content has been practical and useful for addressing the widespread issue of suicide

(STD2)

I wouldn't remove anything. Every module is important

(STD7)

The presentation of content, which included not only textual material but also brief videos, was appreciated as an engaging and practical teaching approach:

The videos are enjoyable, short, and varied

(STD7)

…they even make the course more fun

(STD9)

Students from diverse university disciplines reported no difficulty in understanding the content. They noted that the materials were accessible regardless of their academic backgrounds and suggested that the training should be mandatory for all university students or even society at large. However, they considered it particularly essential for students in health or behavioural sciences programmes such as nursing, medicine, or psychology:

The content is suitable for any student, easy to understand, and you learn a lot

(STD3)

I found the information very relevant and believe it should be taught in all degree programmes

(STD7)

The training enabled students to delve into their understanding of suicide. For many participants, it complemented or built on basic knowledge they had previously acquired. They appreciated that the course went beyond a superficial or introductory level to provide an in‐depth exploration of the phenomenon:

Many training programmes don't delve deeply enough; they only scratch the surface of this issue

(STD9)

I've gained a deeper understanding of the topic and feel that the knowledge I've acquired carries significant responsibility

(STD8)

Participants also expressed satisfaction with the various methods used to assess their acquisition of knowledge, including questionnaires and practical exercises:

The evaluation method was very good

(STD9)

Among all the resources provided during the training, students particularly valued content on appropriate media reporting of suicide, prevalent myths about suicide in society, and available support resources within their community and university institution. Additionally, they praised the optional module on emotional regulation strategies and suggested that it should be included as mandatory content:

I found the journalism activity interesting—how to report on suicide properly and how not to—and debunking myths

(STD3)

I really liked the emotional management module and reading about emotions, anxiety, and emotional avoidance

(STD18)

### 3.3 Perception of Achievements and Expectations

This category captures students’ perceptions regarding the success of the training programme, achieved outcomes, acquired skills, assimilated knowledge, attitudinal changes, and the perceived impact of the educational process upon completing the course.

Participants expressed a sense of group identity and commitment to their role as GUIAS agents for suicide prevention within the university environment:

I now feel comfortable talking about it and informing people, so they know how to approach the topic

(STD6)

We are now more sensitised agents within the university community

(STD5)

Among the most significant achievements, students pointed at the development of reflexivity, critical thinking, awareness, sensitivity, and readiness for suicide prevention. The training allowed them to critically reflect on their past actions and prepare for future improvements, while also enhancing their ability to support and act with individuals at risk of suicide:

I realised I didn't know everything I do now. I can approach things differently in the future—knowing how to listen, what to say, and what not to say

(STD6)

When I encountered friends with this problem before, I didn't know what to say—now I do

(STD6)

Participants gained awareness of the social and political construction of suicide and recognised the urgent need to break stigmas and taboos surrounding suicide and its prevention:

The course helped me realise and confirm that myths, rumours, and poor ways of addressing or discussing suicide exist

(STD17)

There are myths that I didn't even realise were true. I'm pleased to have clarified doubts and societal stigmas that affect us even personally

(STD3)

### 3.4 Suggestions for Improvement

This category includes all recommendations for enhancement or changes to the training programme, both in terms of design and content.

Although participants overwhelmingly felt that the programme exceeded their initial expectations, they suggested expanding on the content to include information and activities focused on other vulnerable groups beyond university students, such as older adults. They also recommended incorporating testimonials from individuals who have survived suicide attempts:

I would like to learn more about other groups, such as older adults

(STD10)

I think it would be helpful to include testimonies from people who have survived suicide attempts

(STD14)

Participants proposed increasing the number of practical in‐person sessions for clinical simulation exercises so that everyone could participate more actively in various scenarios. They emphasised the importance of additional simulation sessions focused on crisis situations involving suicide. Regarding support resources, they suggested introducing new tools such as mobile applications for prevention. They also recommended exploring ways to communicate news about suicide through digital channels like websites or social media platforms rather than solely relying on traditional printed media:

I would have liked more simulation sessions so everyone could participate more

(STD6)

I wish we had learned how to negotiate with someone during a suicide attempt

(STD15)

Finally, participants acknowledged the transversal importance of this training programme for all university students and stressed the need for improved promotional strategies to reach a larger number of students:

Better promotion and dissemination among students are necessary

(STD5)

It needs better promotion next year

(STD3)

## 4 Discussion

The primary objective of the present study was to explore university students’ experiences and perceptions regarding the GUIAS training programme, designed to prepare them as early detection agents and initial responders in suicide risk situations. The findings revealed that the mixed‐method approach, combining theoretical modules, clinical simulations, and reflective activities, offered a flexible learning experience tailored to individual needs and was perceived as helpful to develop preventive competences. This study examined self‐perceived competence only, whereas follow‐up research is needed to assess objective measures and confirm long‐term impact and real‐world applicability of the programme. Participants evaluated the course's accessibility and structure positively, highlighting the role of clinical simulations on improving their practical skills. Furthermore, recommendations for programme enhancements emerged, such as expanding on content and optimising dissemination strategies, underscoring the need to continue improving such initiatives within university settings to strengthen suicide prevention efforts.

The study findings emphasise the importance of designing training strategies within university contexts that integrate flexible and adaptive methodologies, ensuring both accessibility and applicability in suicide prevention. Participants valued the programme's flexibility, which made it easily combinable with other academic and personal commitments. Moreover, evidence suggests that prevention programmes tailored to users’ specific needs are more likely to enhance their effectiveness [27]. In this regard, the ability to access materials at any time and review them according to individual needs supported self‐regulated and efficient learning. Additionally, discussion forums provided a space for exchanging ideas and clarifying doubts, reinforcing the significance of collaborative learning in virtual environments [28].

Another noteworthy aspect is the positive impact of clinical simulation methodology, identified by students as the most effective strategy for comprehending and applying theoretical knowledge. This finding aligns with previous studies that emphasise the utility of simulation in developing mental health competences, as it provides a safe environment for experiential learning and skill development [29].

Participants reported high levels of satisfaction with the relevance and up‐to‐date nature of the topics covered. The usefulness of audiovisual materials was particularly emphasised, with students appreciating their concise and structured format, which helped retain information. Additionally, students perceived the content as applicable across a wide range of disciplines, suggesting that suicide prevention training could benefit not only nursing and psychology students but also those in other health and human behaviour‐related fields [30].

Furthermore, the inclusion of reflective activities enabled students to recognise the influence of myths and stigma associated with suicide. This awareness is crucial, as previous studies [31] have demonstrated that reducing stigma and promoting appropriate communication about suicide can contribute to its prevention. Indeed, the World Health Organisation's fourth strategic recommendation underlines the importance of responsible and effective communication as a key tool in raising awareness and preventing suicide [32]. The activity focused on analysing media coverage of suicide was particularly insightful to encourage critical thinking regarding the portrayal of this phenomenon.

Students reported improved competence in identifying and addressing suicide risk situations. Greater reflexivity regarding past experiences and an active commitment to spread accurate information on suicide were evident. Although these self‐reported gains suggest a positive impact of the training, they do not constitute objective evidence of skill acquisition. Previous studies have shown that training interventions can increase perceived self‐efficacy in crisis management [9, 10]. Future studies should include behavioural assessment tools to evaluate the actual acquisition of skills [33].

The improvement proposals suggested by participants reflect an interest in delving deeper into certain aspects of the course. The inclusion of testimonies from suicide attempt survivors was proposed as a potentially enriching strategy to humanise the issue and foster a more empathetic understanding of individual experiences. Participants also suggested broadening the scope of the course to include other vulnerable groups, such as older adults, indicating the need to adapt the content to encompass a wider range of contexts.

Finally, students emphasised the need to improve the dissemination of the course within the university community, which could enhance its reach and impact. This aligns with previous research demonstrating that effective promotion of prevention programmes is crucial to maximising participation and effectiveness [34].

### 4.1 Limitations, Implications and Proposal for Future Research

This study presents certain limitations that should be considered. Firstly, participant recruitment was conducted through purposive sampling, which may have introduced self‐selection biases, as students with a more positive perception or a greater interest in the topic might have been more likely to participate. In this regard, it would have been valuable to also gather the opinions of those who did not complete the training to better identify potential difficulties or challenges. Secondly, self‐recorded interviews, as opposed to face‐to‐face interviews, may have influenced the depth and spontaneity of participant responses. Additionally, the analysis is based on the transcription of recordings, which implies a potential loss of non‐verbal communicative nuances that could enrich data interpretation. Despite these limitations, the findings offer valuable insights into students’ experiences with the GUIAS training and help identify areas for improvement in future programme implementations, which in turn could be transferred to university contexts with similar characteristics.

The findings of this study have significant implications for the redesign of suicide prevention training programmes in university settings. Specifically, they will allow for the adjustment and contextualisation of future training proposals to enhance their effectiveness, raising awareness and equipping a new generation of students with key skills for the detection, prevention, and management of suicide‐related risks Although the findings of this study could be transferrable to similar university environments, the social, cultural, political, economic and even geographical specifics of each context of implementation must be taken into account in the design of any suicide‐prevention training, as it can significantly influence how university students perceive and engage with such initiatives.

Based on the study findings, several future avenues of research have been identified. Firstly, analysing the perceptions of instructors who delivered the training would be valuable to identify implementation or design challenges and set improvements. Furthermore, longitudinal studies could be conducted to assess the long‐term impact of the GUIAS training, examining whether acquired skills are maintained and put into practice. Future research could also incorporate mixed‐method approaches, combining qualitative and quantitative data to provide a more comprehensive analysis of the programme's effectiveness. Additionally, replicating the study in other universities would help validate the model's transferability and guide the formulation of mental health policies that systematically integrate preventive programmes into curricula. Another promising research direction could focus on evaluating the perception and impact of the training programme among the direct beneficiaries of GUIAS interventions, such as peers or university staff.

Concurrently, and as part of a strategy to reduce stigma and promote participation, suicide prevention content has been integrated into core curricular modules for the 2025–2026 academic year. These include *Psychosocial Health Sciences* (Nursing and Physiotherapy), *Medical Ethics* (Medicine), and *Psychological Assessment* (Psychology). This curricular integration seeks to normalise mental health education and foster broader engagement across the student population [10]. Finally, exploring the integration of advanced digital tools, such as interactive simulations or artificial intelligence, into gatekeeper training could enhance the educational experience for suicide prevention in academic environments.

## 5 Conclusions

This study highlights the suitability of a training programme based on principles of accessibility, active methodologies, and reflective learning for suicide prevention, particularly in the university context. The findings reinforce that the combination of autonomous theoretical‐practical learning and interactive resources appears to be an effective strategy for maintaining motivation and facilitating the acquisition of suicide prevention competences. Furthermore, the study confirms that training in specific suicide prevention skills, such as managing sensitive conversations and recognising warning signs, is crucial in empowering university students as agents of change within their communities.

The improvement suggestions provided by participants offer a solid foundation for future programme enhancements, which could contribute to strengthening mental health training—particularly in suicide prevention—and generating a positive impact both within the university community and society at large.

## Author Contributions

**Patricia García‐Pazo:** conceptualisation (lead), writing – original draft (lead), formal analysis (lead), writing – review and editing (equal). **Paula Nadal Canet:** writing – review and editing (equal). **Margalida Miro‐Bonet:** data curation (lead), Methodology (lead), writing – original draft (equal), writing – review and editing (equal). **Elena Gervilla García:** conceptualisation (supporting), writing – original draft (supporting), writing – review and editing (equal).

## Funding

The authors received no specific funding for this work.

## Ethics Statement

The study was approved by the Ethics Committee at the University of the Balearic Islands (368CER23).

## Conflicts of Interest

The authors declare no conflicts of interest.

## Supporting information

Supplementary Material: Verbatim Interview Transcripts (Original Language).

## Data Availability

The data that support the findings of this study are available on request from the corresponding author. The data are not publicly available due to privacy or ethical restrictions.
