# Supplementary material for: University Students’ Perceptions and Experiences of a Suicide Prevention Gatekeeper Program (GUIAS_Program): Improvements and Implications for Education
Source: Health Expect. 2026 Feb 18;29(1):e70599. doi: 10.1111/hex.70599 (PMC12916852; doi:10.1111/hex.70599)
Supplement: Supplementary file 1 — Supplementary Material: Verbatim Interview Transcripts (Original Language). [file HEX-29-e70599-s001.docx]

Supplementary Material: Verbatim Interview Transcripts (Original Language)

| Themes | Ilustrative quotes |
| --- | --- |
| Satisfaction with the design | 1. “...cada persona puede ir a su ritmo eso facilita compaginar la parte académica con otras cosas...” (STD8). 2. “...es muy útil porque puedes consultarlo todas las veces que necesites...ver los videos varias veces... “(STD1). “...respeta los tiempos de aprendizaje” (STD16).   3. “el curso es muy útil... puedes compartir tus dudas tanto en foro, personas encargadas del curso y compañeros que estudian lo mismo que tú” (STD 1)  4. “...aporta realidad a todos los contenidos teóricos” (STD12), “...los vídeos ... me ha resultado muy práctico verlos aplicados en la simulación clínica” (STD4) |
| Satisfaction with the content | 5. “práctico y útil para la problemática tan extendida como es el suicidio" (STD2), “formación muy buena en todos los sentidos, abarca muchos contenidos" (STD7)  6. "Los vídeos son amenos, cortos y variados" (STD7)  7. “...adecuado para cualquier persona y muy fácil de entender y aprendes un montón” (STD3)  8. “...yo no eliminaría nada...creo que hasta las cosas más prescindibles hacen que el curso sea divertido...” (STD9).  9. “Me ha gustado mucho me ha parecido que la información que tiene es muy relevante y que se debería aplicar directamente en las carreras “(STD7)  10.“Muchas veces siento que cuando nos dan charlas o formaciones de este tipo, no abordan lo suficiente en la temática y solo rascan la superficie de la problemática” (STD9) “He aprendido más a profundidad sobre este tema y siento que el conocimiento que he obtenido tiene una gran responsabilidad...” (STD8)  11.“La forma de evaluar han sido muy buena tanto en los cuestionarios...como en el análisis de las noticias...” (STD9)  12. “m'ha cridat molt l’atenció lo del tema periodístic, com poder comunicar un suïcidi, com se fa, com no i també lo de desmuntar els mites, ho trob super bé” (STD3).  13. “Sobre todo me ha gustado muchísimo la parte de gestión emocional me ha parecido súper interesante leer el tema de emociones ansiedad y evitación emocional” (STD18) |
| Perceived achievements | 14. “Porque ahora como que es algo que me gusta hablar de ello y me gusta también ahora informar un poco a la gente para que sepa porque creo que es importante ... sobre todo aprender cómo abordar el tema” (STD6), “podemos ser por esos agentes un poco más sensibilizados dentro de la comunidad universitaria y la verdad que estaba bastante bien” (STD5)  15. “... me di cuenta que cometí errores porque claro no sabía todo lo que sé ahora y y ahora sí que haría las cosas mucho más diferente de cara a un futuro y a saber cómo escuchar más hay que decir y sobre todo que no decir” (STD6)  16. “…de manera personal cuando me encontraba con amigos míos que tenían este problema no sabía qué decir y ahora ya sí” (STD6).  17. “... ha coincidido la realización de este curso con una muerte por suicidio de un chico he intentado aprovechar para darme cuenta y comprobar que los mitos rumores y malas maneras de dirigirse o de hablar del suceso son una realidad completamente visible” (STD17), “hi ha mites que un mateix pensava que eren així i quan veus la realitat, veus que no estic molt contenta d'aclarir tots els dubtes i els estigmes socials que hi ha hagut que inclus ens repercuteixen a nosaltres mateixos” (STD3). |
| Suggested improvement | 18. “También tocar en profundidad otros colectivos, como personas mayores” (STD10)  19. “tan solo me faltaría complementarlos con el testimonio de una persona que ha sobrevivido a un intento de suicidio” (STD14)  20. “en la parte práctica intentar que participáramos todos un poco más...” (STD6)  21. “Me hubiera gustado saber acerca de cómo negociar con una persona en pleno intento de suicidio, qué debemos hacer en cada caso ... que se puede cometer un suicidio (precipitación, sobreingesta, posesión de armas, etc.)” (STD15)  22. “...més promoció i publicitat en els estudiants, que ojalá a més gent li hagués arribat aquest curs per poder apuntar-se perquè trob que val molt la pena, perquè està super bé” (STD 5), “Sí que me gustaría que este curso se promocionará mucho más de cara al año que viene ...” (STD 3) |
